# Overuse of antibiotics in maternity and neonatal wards, a descriptive report from public hospitals in Dar es Salaam, Tanzania

**DOI:** 10.1186/s13756-021-01014-6

**Published:** 2021-10-09

**Authors:** Mwaka A. Kakolwa, Susannah L. Woodd, Alexander M. Aiken, Fatuma Manzi, Giorgia Gon, Wendy J. Graham, Abdunoor M. Kabanywanyi

**Affiliations:** 1 Ifakara Health Institute, PO Box 78373, Dar es Salaam, Tanzania; 2 London School of Hygiene and Tropical Medicine, Faculty of Epidemiology and Population Health, Keppel Street, London, UK

**Keywords:** Maternal, Neonatal, Antibiotics, Maternity, Tanzania, Stewardship

## Abstract

**Background:**

Overuse of antibiotics is a major challenge and undermines measures to control drug resistance worldwide. Postnatal women and newborns are at risk of infections and are often prescribed prophylactic antibiotics although there is no evidence to support their universal use in either group.

**Methods:**

We performed point prevalence surveys in three hospitals in Dar es Salaam, Tanzania, in 2018 to collect descriptive data on antibiotic use and infections, in maternity and neonatal wards.

**Results:**

Prescribing of antibiotics was high in all three hospitals ranging from 90% (43/48) to 100% (34/34) in women after cesarean section, from 1.4% (1/73) to 63% (30/48) in women after vaginal delivery, and from 89% (76/85) to 100% (77/77) in neonates. The most common reason for prescribing antibiotics was medical prophylaxis in both maternity and neonatal wards.

**Conclusions:**

We observed substantial overuse of antibiotics in postnatal women and newborns. This calls for urgent antibiotic stewardship programs in Tanzanian hospitals to curb this inappropriate use and limit the spread of antimicrobial resistance.

## Background

Overuse of antibiotics is a major challenge worldwide and undermines measures to control antimicrobial resistance (AMR). Failure to control AMR is leading to an increase in mortality, prolonged hospital stays, worsening of clinical conditions and increase in the cost for treatments [1–3]. AMR and specifically, resistance to antibiotic agents for treating bacterial infections, is a major problem in many African countries [4–6]. In Tanzania, an AMR surveillance platform has recently been implemented, but the extent of the burden of AMR currently remains unknown [7, 8]. Overuse (too much use), misuse (wrong use) or high consumption (use of large quantities) of antimicrobial drugs are closely related concepts. Collectively, these are major drivers of antimicrobial resistance [3, 9].

There are multiple reports of high levels of unnecessary prescriptions of antibiotics for both postnatal women, following either cesarean section (CS) or vaginal delivery, and neonates [10–13]. In Sub-Saharan Africa (SSA), although the risk of perinatal infections is high for both mothers and neonates, limited data exists on the use of antibiotics in these groups [14].

Similarly, in Tanzania there are few formal studies of prescribing practices for antibiotics during the postpartum and neonatal period, where maternal and neonatal sepsis are estimated to be responsible for 13% and 20% mortality respectively [15, 16]. As part of a pilot study investigating impacts of an environmental hygiene intervention in Tanzanian hospitals, we aimed to describe antibiotic use in women who recently gave birth and their newborns in three hospitals in Dar es Salaam, Tanzania.

## Methods

### Study design and setting

We conducted point prevalence surveys (PPS) of healthcare-associated infection (HAI) and antibiotic use in maternity, gynecological and neonatal wards as part of the CLEAN study (July 2018–May 2019), a pilot evaluation of an environmental hygiene training intervention [17]. The rounds of the PPS were conducted once a month between October and December 2018. We describe the results of antibiotic use in these hospitals. The surveys were conducted in three public-sector hospitals in Dar es Salaam, Tanzania. Tanzania is an East African country with a population of more than 40 million and is graded lower-middle income by gross national income (GNI), human asset and economic vulnerability development [18, 19]. All three hospitals in this study have separate post-natal wards for vaginal delivery and CS, Hospital 1 and 2 have neonatal wards, while Hospital 3 refers sick newborns to Hospital 2. In 2017, the average number of deliveries per month per hospital was; Hospital 1-1393; Hospital 2-1125 and Hospital 3-1089.

### Data collection procedure

Two doctors from each hospital’s Obstetric Department were trained in how to collect PPS data. They conducted the survey on one weekday per month for three consecutive months, with direct supervision from one investigator (AMK). Data were collected on structured questionnaires adapted from the European Centre for Disease Prevention and Control (ECDC) PPS protocol [20].This tool was originally designed to evaluate the prevalence of HAI in European healthcare facilities [20], and this also captures information on current inpatient antibiotic use. Data were collected at the level of the hospital, ward and individual patient. For individual patients, details of age, sex, date of admission, the presence of an active infection and whether the patient had been given an antimicrobial within the past 24 h of admission were collected. Identifying information including name, address and date of delivery were not collected. These data were extracted from the individual patient case-notes. The reason for prescribing antibiotics was taken from the case notes. If no explanation was provided, it was recorded as “indication unclear”. The paper-based questionnaires were stored in a locked cabinet at the study office. The database was encrypted with a password and only the study data clerk and statistician accessed the data.

### Analysis

Data entry and initial cleaning was done in the HelicsWin.NeT version no 2.4, the recommended software for the analysis of the PPS in the ECDC protocol. Further cleaning and data analysis were conducted in Stata version 14.1. For each ward, the prevalence of antibiotic use was calculated as the percentage of total patients on antibiotics admitted in the ward at the time of the survey.

## Results

### Admission

We collected data from a total of 376 mothers and 162 newborns across three rounds of the PPS in each hospital. Hospital 1 had a total of 173 mothers and 77 newborns, hospital 2 had 258 mothers and 85 newborns, while hospital 3 had 107 mothers only.

### Maternal antibiotic use

The use of antibiotics in CS wards was extremely high: 90% (43/48) of patients in Hospital 1, 96% (45/47) in Hospital 2 and 100% (34/34) in Hospital 3. In Hospital 1, all mothers with suspected infections were transferred to the gynecology ward, thus all the antibiotics prescribed in CS ward were for post-operative surgical prophylaxis. At Hospital 2, only four out of 45 mothers on antibiotic treatment were reported to have active infection; one with deep surgical site infection (SSI) (first survey) and three with superficial SSI (third survey). All other women (91%; 41/45) were receiving antibiotics for post-operative surgical prophylaxis. In Hospital 3, all admitted mothers (100%; 34/34) were on antibiotics. One SSI was reported in the first survey, but all other antibiotics were prescribed for surgical prophylaxis (see Table 1). In the post-natal vaginal delivery wards, antibiotic use varied considerably between hospitals and over time. The overall use of antibiotics was 63% (30/48) at Hospital 1, 11% (14/126) at Hospital 2, and 1.4% (1/73) at Hospital 3. At Hospital 1, use of antibiotics increased from 18% (2/11) in the first survey to 100% (22/22) in the third survey; all antibiotics were prescribed for medical prophylaxis. At Hospital 2, 11% (14/126) were prescribed antibiotics. Out of these, 7% (1/14) were for hospital-acquired episiotomy infection, 7% (1/14) were for community-acquired infection, 57% (8/14) were for medical prophylaxis and the remainder 29% (4/14) were without clear indications. At Hospital 3, only one woman (1.4%;1/73) was receiving antibiotics which was for surgical prophylaxis (see Table 2.)

**Table 1:** Prevalence of antibiotic use on caesarean section wards

Hospital	Survey round	Total mothers	Mothers on antibiotics n (%)	Reason for antibiotic use out of all mothers on antibiotics n (%)			
				Community-onset infection	Hospital-acquired infection	Medical/surgical prophylaxis	Unclear indication
Hospital 1	1	16	15 (93.8)	0	0	15 (100)	0
	2	15	11 (83.3)	0	0	11 (100)	0
	3	17	17 (100)	0	0	17 (100)	0
Total		48	43 (89.5)	0	0	43 (100)	0
Hospital 2	1	17	16 (94.1)	0	1 (6.3)	15 (93.7)	0
	2	15	14 (93.3)	0	0	14 (100)	0
	3	15	15 (100)	0	3 (20)	12 (80)	0
Total		47	45 (95.8)	0	4 (8.9)	41 (91.1)	0
Hospital 3	1	11	11 (100)	0	1 (9.1)	10 (90.9)	0
	2	11	11 (100)	0	0	11 (100)	0
	3	12	12 (100)	0	0	12 (100)	0
Total		34	34 (100)	0	1 (2.9)	33 (97.1)	0

**Table 2:** Prevalence of antibiotic use on post-natal wards

Hospital	Survey round	Total mothers	Mothers on antibiotics n (%)	Reason for antibiotic use out of all mothers on antibiotics n (%)			
				Community onset infection	Hospital acquired infection	Medical/surgical prophylaxis	Unclear indication
Hospital 1	1	11	2 (18.2)	0	0	2 (100)	0
	2	15	6 (40.0)	0	0	6 (100)	0
	3	22	22 (100)	0	0	22 (100)	0
Total		48	30 (62.5)	0	0	30 (100)	0
Hospital 2	1	36	6 (16.7)	1 (16.7)	1 (16.7)	4 (66.6)	0
	2	45	4 (8.9)	0	0	4 (100)	0
	3	45	4 (8.9)	0	0	0	4 (100)
Total		126	14 (11.1)	1 (7.1)	1 (7.1)	8 (57.2)	4 (28.6)
Hospital 3	1	20	1 (5)	0	0	1 (100)	0
	2	26	0	0	0	0	0
	3	27	0	0	0	0	0
Total		73	1 (1.4)	0	0	1 (100)	0

### Neonatal antibiotic use

All newborns (77/77) in Hospital 1 were receiving antibiotics; 17% (13/77) had HAI, 1.3% (1/77) had community-acquired infection, and the rest (82%; 63/77) were being given antibiotics for medical prophylaxis. In Hospital 2, 89% (76/85) were receiving antibiotics across all three rounds of the survey, increasing from 80% (24/30) during the first round to 100% (28/28) in the third round. Out of 76 newborns on antibiotics, 4% (3/76) had community-acquired infections, 40% (30/76) had HAI and 57% (43/76) had no clear indication for antibiotic use (see Table 3).

**Table 3:** Prevalence of antibiotic use on neonatal wards

Hospital	Survey round	Total Neonates	Neonates on antibiotics n (%)	Reason for antibiotic use out of all neonates on Antibiotics n (%)			
				Community onset infection	Hospital acquired infection	Medical/surgical prophylaxis	Unclear indication
Hospital 1	1	33	33 (100)	1 (3.0)	13 (39.4)	19 (57.6)	0
	2	26	26 (100)	0	0	26 (100)	0
	3	18	18 (100)	0	0	18 (100)	0
Total		77	77 (100)	1 (1.3)	13 (16.9)	63 (81.8)	0
Hospital 2	1	30	24 (80.0)	1 (4.2)	11 (45.8)	0	12 (50)
	2	27	24 (88.9)	1 (4.2)	9 (37.5)	0	14 (58.3)
	3	28	28 (100)	1 (3.6)	10 (35.7)	0	17 (60.7)
Total		85	76 (89.4)	3 (4)	30 (39.5)	0	43 (56.5)

There was no inpatient neonatal ward in Hospital 3 at the time of survey

## Discussion

We performed repeated rounds of PPS for antibiotic use in the maternity and neonatal departments of three busy public sector hospitals in urban Tanzania. We found high levels of antibiotic use in women following vaginal delivery or caesarean section. Around 95% of surveyed women who had CS were receiving antibiotics, and among these, 95% were receiving them for post-operative surgical prophylaxis lasting more than one day. The high use was observed in all three hospitals and in all surveys. Prescription of antibiotics in post-natal wards varied widely between hospitals: while Hospital 1 prescribed antibiotics in about 60% of women, Hospitals 2 and 3 only prescribed 11% and 1.4% respectively. The most common reasons for antibiotic prescription were medical or surgical prophylaxis in all hospitals. Similarly, we observed high use of antibiotics, at around 90%, in neonatal wards, with the vast majority being used for medical prophylaxis or without a clear indication. This near-universal prescription of antibiotics after CS is a common clinical practice in low income countries [21–23], despite the absence of evidence or recommendations to support it. By contrast, in high income countries, the irrational use of antibiotics after CS and in maternity wards is reported to have dropped markedly over recent years [10]. This variation in practice may be explained by the onset of antibiotic stewardship programs in many hospitals in high-income countries [24]. In hospitals with limited resources, prescribing post-operative prophylactic antibiotics is likely to be influenced by concerns about high local rates of SSI, in turn attributable to weak measures for infection prevention and poor hygiene in surgical environments [25]. However, to the best of our knowledge there is no evidence that post-operative antibiotic prophylaxis helps to prevent surgical site infections. In Tanzania, infection prevention and control (IPC) data from health facilities indicates low adherence to IPC guidelines among health care workers [25, 26]. In this study, antibiotics were prescribed to one quarter of women with vaginal delivery, with substantial variation between the facilities. The highest use was reported in Hospital 1 (62.5%), most commonly for prophylaxis, whereas antibiotic use was relatively low for women following vaginal delivery in Hospital 2 (11.1%) and Hospital 3 (1.4%). We believe this wide variation between hospitals is likely to reflect differences in prescribing practices between the facilities or individual health care workers, rather than differences in infection rates, though we cannot conclusively confirm this. In other low-income settings, high use of antibiotics in women with vaginal delivery has been reported in India and Vietnam, and in a WHO study combined data from Africa, Asia and the Americas [12, 23, 26].While the study in India reported the lack of local guidelines at the time of the study as a possible factor underlying the high antibiotic prescribing rate, it was not clear whether the Vietnamese and WHO studies were in facilities with availability of guidelines for management. On the other hand, studies from Sweden and USA have reported a low use of antibiotics after vaginal delivery, reflecting the well-established nature of antibiotic stewardship programs in these countries [10, 23, 28].

In this study, we expected to see a difference in antibiotic use between the CS and vaginal delivery because CS carries higher risk of infection. Prophylactic antibiotics are recommended in CS but they should be given pre-rather than post-operatively. Irrational overuse of antibiotics is likely to ultimately lead to severe illness and death in mothers and newborns as a result of increasing levels of antibiotic resistance in low-income countries [3]. More than 90% of the admitted neonates were given antibiotics during this survey, typically as medical prophylaxis or without a clearly recorded indication. The wards were staffed by pediatricians and general practitioners, and it is possible that prescribing practice would be different among specialist neonatologists. However, specialists are rare for regional hospitals in Tanzania, so we believe our findings would be generalizable to other hospitals in Tanzania. Similarly, high use of antibiotics in neonates has been reported in one study in India where around 90% of the admitted neonates were given antibiotics [27]. Use of antibiotics in high-income countries is reported to have dropped significantly in the past years among neonates [28]. Early neonatal antibiotic exposure may be associated with morbidities such as allergy, obesity, gastrointestinal disorders and acquisition of carriage of antibiotic resistant bacteria, though the underlying evidence is weak [29–31].

### Study limitations

The findings in this study might have been subjected to a form of Hawthorne effect since the clinicians involved in the data collection were also working on the maternity wards and prescribing antibiotics to postnatal women. It is therefore possible that performing the survey increased their concern about infections and could have influenced their prescribing practice in later rounds of the survey. This may partly explain the increase in prescribing seen at Hospital 1 during the course of the study.

## Conclusion

There was an extremely high prevalence of antibiotic prescribing for prophylaxis in maternal and neonatal units in hospitals in Dar es Salaam, Tanzania. Better infection control policies, local and national guidelines, and antimicrobial stewardship programs need to be implemented to avoid unnecessary prescription of the antibiotics and promotion of antibiotic resistance.

## Data Availability

Data and materials of the study will be available on request to the corresponding author.

## Abbreviations

AMR: Antimicrobial resistance
CS: Cesarean section
ECDC: European center for disease prevention and control
GNI: Gross national income
HAI: Healthcare associated infection
IPC: Infection prevention and control
PPS: Point prevalence survey
SSA: Sub-Saharan Africa
WHO: World health organization
